# From Passive Patient to Engaged Partner: My Journey With Parkinson Disease

**DOI:** 10.2196/12566

**Published:** 2020-04-16

**Authors:** Richard Higgins, Maureen Hennessey

**Affiliations:** 1 Precision for Value New York, NY United States

**Keywords:** Parkinson disease, Parkinson disorder, parkinsonism, movement disorder, physical exercise, aerobic exercise, physical activity, running, race-walking, patient self-care, patient self-advocacy, patient self-efficacy, coaching, health coaching, care management, patient engagement, shared decision making, participatory medicine, patient-centered care, patient-centered outcomes, caregiver support, positive psychology, Parkinson disease quality measures, quality management

## Abstract

This patient narrative by Richard Higgins with Maureen Hennessey describes Richard's journey of learning to live with a chronic and progressive illness. It begins with Richard's diagnosis and shares many of the lessons learned along the way. Richard copes daily with this condition, relying on the support and expertise of his wife and the treatment team he has assembled while also encouragingly drawing on the skills and knowledge gained as a longtime running coach. A clinical commentary is provided at the article's conclusion, drafted by Richard's friend, Maureen Hennessey, PhD, CPCC, CPHQ, offering observations about the relevance of Richard's story to participatory medicine and suggesting pertinent resources for patients and health care professionals.

## Patient Essay by Richard Higgins

### My Diagnosis

In the spring of 2009, after falling down the stairs several times within a week, I knew something was wrong. I needed to find out what it was. I received an initial diagnosis of Parkinson disease (PD) and was referred to a movement disorder specialist for confirmation. They said it was not PD and sent me back to the first neurologist. He then gave me a tentative diagnosis of a parkinsonism.

Next, I was sent to a Movement Disorder Center where, more than 2 years and 4 doctors into this journey, I was diagnosed with PD. The parkinsonism that was earlier suspected typically progresses much faster and does not respond as well to current treatments, so my wife and I were relieved by the diagnosis. Then, the question became, what to do about it?

PD includes an array of symptoms beyond the tremors, shaking, and shuffling steps that are familiar to the general public. Symptoms vary in sequence and severity for every person. These include stiffness and rigidity, inability to reflect moods on your face, reduced fine motor coordination, disrupted sleep, decreased sense of smell, balance issues, mild cognitive impairment, depression, poorer/smaller handwriting, delayed reaction time, and difficulty swallowing. Like many chronic conditions, medications have helped with some symptoms, but do not cure or retard the progress of this illness.

Prior to my diagnosis, I had always taken a passive role in my health care. Not being a doctor or clinician, I have been accustomed to doing what physicians tell me to do or, in less serious matters, following suggestions in various fitness articles. Yet, due to the symptom complexity and variability of PD, it became clear that no single doctor could manage all of my care or could know everything I needed to do to manage my condition.

### Becoming an Engaged Participant in My Care

It was good news when my neurologist mentioned that intensive aerobic exercise has been shown to slow progression of PD, since I enjoy running and race-walking. My experience as a cross-country and track and field coach helped me tap into my knowledge of goal-setting, physical activity, and planning workouts to help not only my students but also myself.

In the ensuing years, I tailored my training plan to try to minimize the impact of PD for as long as possible. I transitioned from being serious about my workouts, especially my cardio sessions, to pushing myself to a nearly fanatical degree. I was able to incorporate various exercises and techniques from specialists I consulted (such as physical therapists, chiropractors, and strength and conditioning coaches) into my routines. I created a plan that I could modify as my condition changed and as I learned new things. This plan now includes a combination of aerobic and cardio intensity, balance exercises, core conditioning, general body strength, and fine motor coordination. As I learn and my efforts evolve, several important concepts have emerged:

1. No matter the stage of PD, some type of exercise is beneficial. Muscles atrophy quickly if not used. Muscles that are used degenerate more slowly.
2. Be involved in, and committed to, your training plan. While sometimes it can be a struggle even to do the routines you enjoy, planning your workouts helps you “buy in” to them more completely.
3. Incorporate enjoyable activities into your plan. It’s easier to stay with a routine—particularly one you need to do long-term—the more you enjoy it. This can also provide some feeling of control with an unpredictable disease.
4. Begin slowly. Increase the amount, types, and intensity of exercises just a little each week. Remember: always discuss any plans for physical activity with your physician(s).
5. Learn what exercises are within your ability and which can be beneficial. With any chronic condition, one exercise routine does not fit all (especially with PD). Establish baselines, set goals, and measure progress.
6. Take advice from a variety of professionals. Over time, professionals such as occupational therapists, psychologists, athletic trainers, and aging specialists may become part of your treatment team. Candor about your condition, including your symptoms, reaction to drugs, and amount you exercise, helps your clinicians to better help you. It may help if you provide them with information before appointments so they are prepared, but not all clinicians will be able to work that way.
7. Include caregivers early. I also think of my wife as my “care-partner.” Caregivers or care-partners can help you solve problems in adapting to living with PD, be a coadvocate for you, and provide information to your clinicians that you may forget. It will also make it much easier for them to transition into this role.

### Learning to Ask for Help: Lessons in Grace and Humility

This last point—knowing the need for and accepting a caregiver—has been critical in handling the struggle with PD. As my symptoms gradually worsen, I have had to accept that there are some things I just can’t do safely. Tasks to avoid, like washing sharp knives or crystal glasses, dawned on me long after becoming obvious to my wife. Unfortunately, it took much longer to realize that my reluctance to accept her help only increased her burden. Not communicating to her my symptoms, frustrations, and needs (which I am still trying to perfect) can cause her to expend more energy and time. As the PD continues to progress, she has gradually assumed more of a care-partner role. There is added urgency now for me to talk with her about my successes, failures, and plans. When she knows what is happening and what I am doing, she is better able to help me coordinate my care, rather than having to guess what I really want or need.

Surprising myself, I decided to give up driving. After developing a delayed reaction in my right foot and leg, I realized that having any hesitancy while operating a vehicle could prove disastrous. Unfortunately, this also led to the realization that I needed help from other people to get where I had to go. I resented this loss of freedom, as well as having to ask for help, accepting it when it was offered, and being grateful for it. I have had no difficulty finding people to drive me and am indebted to my wife and daughter, as well as to fellow coaches and parents of cross-county team members. But it has been difficult admitting (even to myself) that I need assistance. This has improved over the last several years but is always hard. When I decided to approach this challenge the way I approached my exercise routine, I began setting goals for communicating my needs and expressing appreciation for the support. This has helped me maximize my independence while also accepting my limitations with grace.

### More Lessons Learned: The Journey is Always Evolving

I’m now in the ninth year of my journey. There have been many joyous milestones (graduations, a son’s wedding, a daughter’s wedding, and the birth of grandchildren) that make me realize how important it is to do all I can in this progressive fight. There have been exciting times watching students win cross-country and track championships and going on wonderful trips with my wife. However, I also must learn to deal with setbacks. Any plan, no matter how well thought out, can go awry at times. One winter, I was not prepared for just such a setback. A required minor surgery prevented me from exercising for nearly 4 months. Not only did I lose a lot of conditioning, but also a routine I depended on psychologically. I experienced some changes in physical symptoms, some loss of cognitive ability, and signs of depression. When I was able to exercise again, I also had to work on my mental and emotional conditioning. I have come back somewhat, but not yet to presurgery levels.

I have learned a great deal throughout this journey. First and foremost, to remain as active as possible, so I can enjoy more great moments. I have learned there always will be unavoidable challenges. For example, during the last few months, I have undergone additional testing. This has revealed several atypical indicators and symptoms, suggesting the possibility that my PD condition may present atypically or could perhaps be another parkinsonism. Depending on what is ultimately learned, my treatment and self-care may require more adjustments.

While there are many advisors who can help during those times, I have learned that my caregiver(s) and I are the people who can best manage this team. I have learned to be involved in my care and graciously allow others to help when needed while continuing to do the things I can do. I am still learning these things, and it is not always easy. Planning with PD is tough, as my symptoms change from day-to-day and even throughout the day. It helps to prioritize exercise and to focus on the importance of enjoying life while coping with this difficult condition. Although there is no cure for PD yet, I continue to be hopeful about the many new treatments to help manage it. Overall, staying active through exercise and being involved in my care helps me be more positive about today and the days to come.

## Invited Clinician Comment by Maureen Hennessey

This narrative describes a patient’s journey of learning to live with a chronic and progressive illness. It begins with the diagnosis, moves through patient participation, and emerges with many lessons learned along the way. Coping daily with this condition, he relies on the support and expertise of a spouse and other family members, and the treatment team assembled, while encouragingly drawing on the skills and knowledge gained as a longtime running coach. His story exemplifies a key concept in participatory medicine: all of us must be participants in and contribute to our care, to the extent of our preferences and abilities. We can all admire his courage, candor, and tenacious self-care, exemplified by his thoughtful approach of seeking and accepting assistance and support. As clinicians, care managers, health coaches, and health quality experts, we can learn from descriptions of how patients and caregivers collaborate and partner with each other and with health care professionals to seek and use information resulting in well-informed personal choices. Patient goal-setting, problem-solving, and positive psychology are instructive, particularly as patients strive to enjoy life and help others while hoping for better treatments to manage their conditions.

At least 10 million individuals worldwide, including more than 1 million Americans, live with PD [1]. For additional patient and caregiver education and resources, and information about finding a cure, you can visit the American Parkinson Disease Association [1] website and the Michael J. Fox Foundation for Parkinson's Research [2] website. Information about quality measures pertinent to the treatment of PD (including exercise, mood, and rehabilitation) may be found in “Quality improvement in neurology: Parkinson disease update quality measurement set” on the American Academy of Neurology website [3].

## Abbreviations

PD: Parkinson disease
